# Supplementary material for: Conformational Transition of Semiflexible Ring Polyelectrolyte in Tetravalent Salt Solutions: A Simple Numerical Modeling without the Effect of Twisting
Source: Int J Mol Sci. 2024 Jul 29;25(15):8268. doi: 10.3390/ijms25158268 (PMC11311450; doi:10.3390/ijms25158268)
Supplement: Supplementary file 1 [file ijms-25-08268-s001.zip › ijms-3056759-supplementary.pdf]

Supporting Information

Conformational Transition of Semiflexible Ring Polyelectrolyte in Tetravalent Salt Solutions

Dan Lu[1], Ai Hua Chai[2], Xiu Xia Hu[1], Peihua Zhong[3], Nian Qian Kang[1], Xian Fei Kuang[1], and Zhi Yong Yang[1,*]

[1] Department of Physics, Jiangxi Agricultural University, Nanchang 330045, Jiangxi, China
[2] College of data sience, Jiaxing University, Jiaxing 314001, China
3 College of computer information and engineering, Jiangxi Agricultural University, Nanchang 330045, Jiangxi, China.
* Correspondence: zyyang@jxau.edu.cn(Z.Y.-Y).

This supporting information contains 2 Figures over 2pages

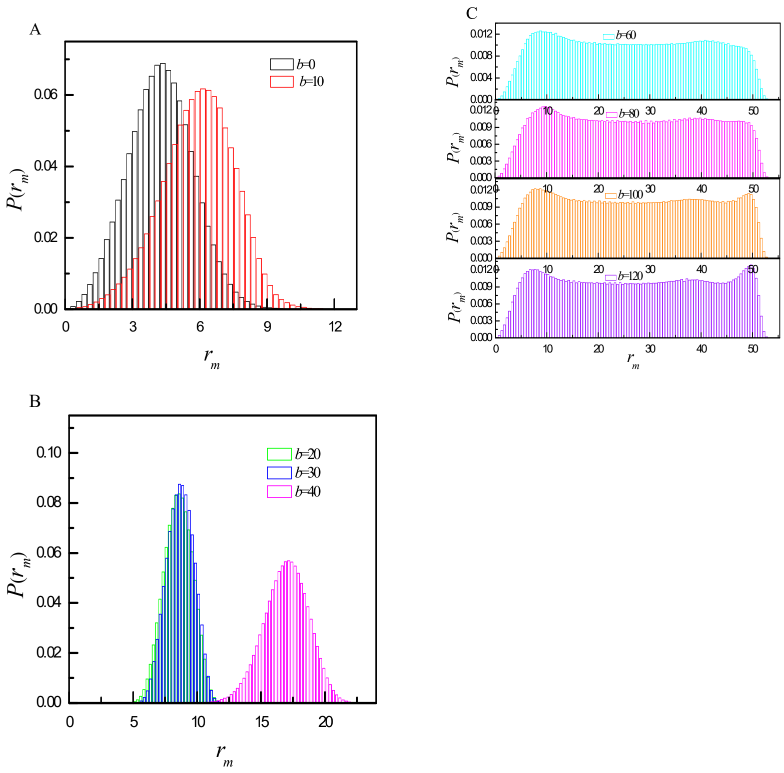

Figure S1. The number density of monomers $P(r_m)$ as a function of the distance r_m from the center of mass of ring PE for different bending energy b at C_S=1.78×10^{-3}M.

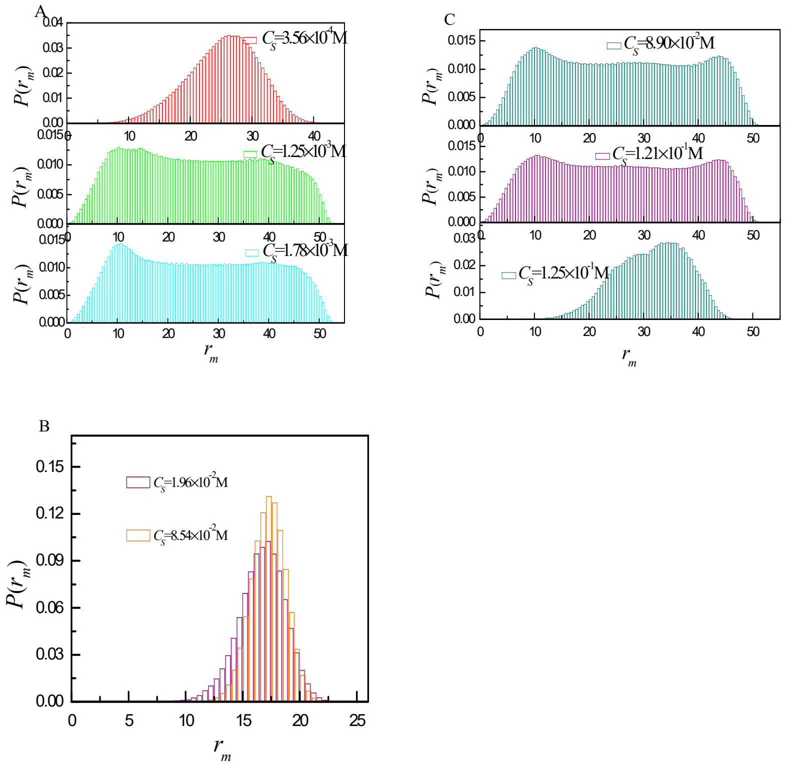

Figure S2. The number density of monomers $P(r_m)$ as a function of the distance r_m from the center of mass of ring PE at different salt concentration C_S for b=60
